# Undergraduate students’ choices around community service and internship: A single faculty study

**DOI:** 10.4102/sajp.v80i1.1980

**Published:** 2024-04-29

**Authors:** Heather Talberg, Tamara Dayaram, Sarah Geel, Sivuyile Mthembu, Rofhiwa Nthangeni, Savannah Pillay, Theresa L. Burgess

**Affiliations:** 1 Department of Health and Rehabilitation Sciences, Faculty of Health Sciences, University of Cape Town, Cape Town, South Africa; 2 Centre for Medical Ethics and Law, Faculty of Medicine and Health Sciences, Stellenbosch University, Stellenbosch, South Africa

**Keywords:** undergraduate training, internship, community service, professional support, MBChB, health and rehabilitation

## Abstract

**Background:**

The Internship and Community Service Programme (ICSP) places newly graduated health professionals for a compulsory training period. Universities adopt multiple strategies to encourage students to select rural placements for ICSP.

**Objectives:**

This study describes ICSP choices among final-year MBChB and Health and Rehabilitation Science students at a South African university and the factors influencing their decisions.

**Method:**

A cross-sectional qualitative descriptive study was conducted using a self-developed online questionnaire. Eighty-five final-year students were recruited.

**Results:**

Most respondents (*n* = 38, 45.8%) chose the Western Cape (WC) as their first choice placement. There was a significant difference between MBChB and other health science students’ choice of level of healthcare (χ = 10.39, *p* = 0.006), with MBChB less likely to choose primary healthcare (PHC) level placements. District and tertiary care options were perceived as better sites for learning and practice, while PHC and rural sites, considered underresourced and understaffed, were avoided. Although MBChB students indicated a lower preference for rural placements, this was not significant.

Factors influencing ICSP application decisions included professional support, work environment, and social and personal considerations.

**Conclusion:**

Students’ placement choices were based on their perceptions of clinical exposure, learning opportunities, mentorship and supervision. Placements closer to home were preferred. UG clinical exposure and rural background seem to have some impact on choice but need further investigation.

**Clinical implications:**

Universities should continue to explore ways to improve students’ readiness for practice in underresourced settings. Improved exposure to rural and PHC sites during training could encourage better uptake during ICSP placement.

## Introduction

The Internship and Community Service Programme (ICSP), introduced by the South African National Department of Health in 1998, enables newly qualified healthcare professionals, including doctors (MBChB) and Health and Rehabilitation (HR) professionals, including occupational therapists (OT), physiotherapists (PT), speech-language therapists (SLP) and audiologists (AUD), to further their professional training through a mandatory year of clinical practice (Sein & Tumbo 2012). The ICSP further aims to strengthen the public healthcare system by overcoming the human resource challenges faced in particular in rural and peri-urban areas in South Africa (Harrison 2009; Maseko et al. 2014; Nadasan & Chetty 2020; Van Stormbroek & Buchanan 2016). On completion of the ICSP, graduates may be registered with the Health Professions Council of South Africa for independent professional clinical practice (Department of Health 2020a).

The location of health science graduates’ placement for their ICSP largely depends on their application for preferred ICSP posts during their final year. Through an online system, students select five preferred sites that must be spread across different healthcare levels and at least three provinces (Department of Health 2020b). The Department of Health necessitates applicants to choose facilities based on priority rankings, determined by whether they are an urban, peri-urban or rural facility and staffing levels (Department of Health 2020b).

Various efforts have been made by the South African National Department of Health together with universities to motivate health science graduates to choose to practice in rural settings for the ICSP and beyond, including clinical exposure to rural sites and recruitment of students from rural areas (Budhathoki et al. 2017; McAuliffe & Barnett 2010; Ray, Young & Lindsay 2018; Van Stormbroek & Buchanan 2016). However, multiple studies have demonstrated that health science students favour urban healthcare facilities (Daniels-Felix, Conradie & Voss 2015; McAuliffe & Barnett 2010; White & Humphreys 2014).

At the University of Cape Town (UCT), students in the Faculty of Health Sciences (FHS) from the MBChB programme as well as the Department of Health and Rehabilitation Sciences (DHRS) complete multiple prescribed clinical rotations in preparation for independent clinical practice and to meet the Health Professions Council of South Africa training requirements. These clinical rotations expose students to the scope of their chosen profession and the various levels of healthcare and social contexts in which professional practice occurs.

With supporting evidence from Gilson and Erasmus (2005), Maseko et al. (2014) identified various factors influencing the choice of community service placements among occupational therapy students within the Gauteng and Limpopo provinces. However, there is limited general research on the factors that influence the choice of placement for community service or internship among health science students in South Africa.

### Objectives

This study aimed to describe the choices of ICSP among a group of final-year healthcare students in both MBChB and the HR Sciences of PT, OT, SLP and Aud at a South African University and the broad factors which may influence their decisions.

## Research methods and design

### Design

The study had a descriptive cross-sectional quantitative design using a self-developed questionnaire.

### Participants

All undergraduate final-year FHS students who were expected to graduate and enter the ICSP in 2022 were recruited for the study during the 2021 academic year. There were approximately 400 registered undergraduate final-year students. Based on the population size of 400, with an acceptable margin of error of 5% and confidence levels of 80%, a sample size of 116 participants was calculated using Epi Info (Version 7.2.4.0) StatCalc for population surveys or descriptive studies.

### Main outcome measures

The final-year physiotherapy UG student researchers developed the online questionnaire in consultation with the study supervisors. The questionnaire comprised both closed and open-ended questions. In Section A, respondents’ demographic information was requested. In Section B, the factors that influenced their placement choice for the ICSP were considered and rated using a Likert scale. Open-ended questions provided follow-up and allowed for clarification of the respondent’s choices and influences.

The questionnaire was validated by two validators (Appendix 1). The validators were a final-year Master’s degree student in the DHRS and a part-time lecturer in the Department of Health Sciences Education. Both had developed questionnaires as part of their studies and were familiar with the validation process and requirements. The first draft of the survey was distributed to the validators in the form of a Microsoft Word document to allow comments to be made on the survey. The validators provided feedback on both the content and face validity and appropriateness of the questions in the survey, in line with the study objectives. The changes suggested by the validators were implemented. The survey was then formatted on the LimeSurvey online programme. The LimeSurvey link was shared with the validators to ensure that the link worked and to make any final changes to the questions before commencing the pilot study. The pilot study participants consisted of two MBChB students and two of each DHRS discipline, with 10 students recruited. The researchers emailed the students with a link to the questionnaire. They were required to complete informed consent before participation. The pilot aimed to identify potential question misinterpretations, the time needed to complete the questionnaire, and any errors with the questionnaire itself. Pilot data were included in the final study analysis as no significant changes were needed. The students had 1 week to answer the survey and provide feedback. Based on unexpected changes to the cost and usability of LimeSurvey for the researchers, the survey was exported to Google Forms for use in the study. This necessitated minor layout changes to the survey. All survey questions were compulsory.

### Recruitment

Institutional approval was obtained from the University’s Department of Student Affairs (DSA) to recruit students for the study. All final-year FHS students were recruited to take part in this study. Information on the research and a link to the questionnaire were sent to each discipline’s class representatives and administrators for distribution to the larger cohort. Due to ongoing COVID-19 related restrictions, no face-to-face recruiting was possible. All participants had to provide informed consent before gaining access to the questionnaire. Data were collected anonymously. Students who did not provide consent were excluded from the final study sample. The questionnaire was available online for 6 weeks at the beginning of the 2021 second semester. Reminders were sent on four occasions over the period to encourage participation.

### Statistical analyses

The data from Google Forms was imported onto a Microsoft Excel spreadsheet for analysis. Data were analysed using SPSS statistical software (IBM Corp. Version 20.0). Descriptive data are presented as numbers and percentages. A Pearson’s chi-square test was used to determine differences between categorical values. Statistical significance was accepted as *p* < 0.05. Open-ended responses were grouped using an inductive approach to content analysis, and trends were identified (Singer & Cooper 2017). These were discussed and confirmed by the student researchers and supervisors.

### Ethical considerations

This study was approved by the Human Research Ethics Committee at UCT (HREC Ref: 167/2021). It was guided by the Declaration of Helsinki’s ethical principles (World Medical Association 2001). Institutional approval to recruit students was obtained from the University’s DSA. All participants had to complete written informed consent before accessing the survey and had the right to withdraw from the study at any point. Information was collected anonymously, and the student researchers were not eligible to participate. Data were securely stored in a cloud-based folder, only accessible to the research team.

## Results

### Participants

Of the 400 registered final-year FHS students at UCT, 85 respondents completed the questionnaire (Table 1). No respondents were excluded from the study. Where appropriate, results were analysed to reflect comparisons between students in MBChB and DHRS. No analysis according to specific professional discipline was possible due to a smaller than anticipated sample size.

**TABLE 1:** Demographic profile of participants (*N* = 85).

Demographic variables	*n*	%
**Gender**		
Female	71	83.5
Male	12	14.1
Other	2	2.4
**Age (years)**		
21	9	10.6
22	24	28.2
23	20	23.5
24+	32	37.7
**Marital status**		
Long-term relationship	27	31.8
Married	3	3.5
Single	55	64.7
**Department of study at UCT**		
MBChB	40	47.1
DHRS	45	52.9

UCT, University of Cape Town; DHRS, Department of Health and Rehabilitation Sciences; MBChB, Bachelor of Medicine, Bachelor of Surgery.

The majority of students originated from the Western Cape (WC) (*n* = 43, 50.6%), followed by Gauteng (*n* = 17, 20%) and KwaZulu-Natal (*n* = 9, 10.6%). A small representation occurred from all other provinces. Eighty-four percent of students (*n* = 71) indicated that they were from an urban setting in their home province, as opposed to a rural (*n* = 2, 2.4%) or peri-urban (*n* = 12, 14.1%) setting. Sixty percent of students (*n* = 51) indicated English as their home language, followed by Afrikaans (*n* = 11, 12.9%) and IsiZulu (*n* = 8, 9.4%). Six other African languages were indicated as the home language for the remainder of the students (*n* = 15).

### Choice of province for internship and community service programme

The students’ ICSP first choice (*n* = 84) provinces were WC (*n* = 38, 45.8%), followed by KwaZulu-Natal (*n* = 17, 20.2%) and Gauteng (*n* = 14, 16.7%). Limpopo, North-West and the Northern Cape were each selected by only one student as their preferred first choice. Of the 43 students originating from the WC, 28 (65.1%) selected the province as their first choice. Moreover, 90.7% of these students indicated it as a placement choice. This shows a strong correlation between students from the WC selecting their home province for their ICSP placement (χ = 44.739, *p* = 0.006).

Students also indicated their preferences for the level of healthcare for their ICSP placement. There was a significant difference between MBChB and DHRS students’ choice of level of healthcare for the ICSP placement (χ = 10.39, *p* = 0.006) (Table 2).

**TABLE 2:** Student preferences for the level of healthcare for internship and community service programme placement (*N* = 85).

Department of study	Level of healthcare							
	Primary healthcare level or clinic or CHC (*n*)	%	District or regional hospital (*n*)	%	Tertiary hospital (*n*)	%	*n*	%
MBChB	1	1.2	30	35.2	9	10.6	40	47.1
DHRS	11	12.9	21	24.7	13	15.3	45	52.9

**Total**	**12**	**14.1**	**51**	**59.9**	**22**	**25.9**	**85**	**100**

MBChB, Bachelor of Medicine, Bachelor of Surgery; DHRS, Department of Health and Rehabilitation Sciences; CHC, Community Healthcare centres.

Students expanded on some reasons for their choices, considering both perceived professional support structures and personal or social support at placements. District and regional facilities seemed to provide a balance between supervision and opportunities for growth as well as learning from a range of cases:

‘It has a balance of both complex and standard care patients.’ (MBChB, male, 6th year)

‘I think it will be the best of both worlds in terms of exposure and knowledge and experience gained.’ (DHRS, female, 4th year).

The students’ experiences during clinical placements were also a consideration:

‘I have worked at clinics for most of my clinical experience so far and enjoy the work environment and the experience that one gain.’ (DHRS, male, 4th year).

Sixty percent of DHRS students (*n* = 27) indicated they would consider rural placement for community service. Although MBChB students showed a lower preference for rural placements, there was no significant difference between MBChB and DHRS students (Table 3).

**TABLE 3:** Students’ preferences for rural placement for the internship and community service programme (*N* = 85).

Department of study	Yes (*n*)	%	No (*n*)	%	Undecided (*n*)	%
DHRS	27	60.0	10	22.2	8	17.8
MBChB	15	37.5	17	42.5	8	20.0

**Total**	**42**	**49.4**	**27**	**31.8**	**16**	**18.8**

DHRS, Department of Health and Rehabilitation Sciences; MBChB, Bachelor of Medicine, Bachelor of Surgery.

The reasons in support of rural placements ranged from the acknowledgement of their home background:

‘I was born in a rural area and would love to go back.’ (MBChB, female, 6th year)

To the recognition of areas of need and opportunities to broaden their professional outlook:

‘I would love to go to a rural placement because I have always lived in suburban areas. Experiencing how other people live and different cultures, contexts and languages is crucial in South Africa.’ (DHRS, female, 4th year)

Healthcare disparities were acknowledged in thinking around rural placements:

‘Rural areas are places that benefit from the community service community, and it’s where most help is needed, and many professionals do not want to go there.’ (MBChB, female, 6th year)

‘I feel that the under-resourced areas are more in need of my services.’ (DHRS, male, 4th year)

Of the students who were against or undecided on rural placements, some did acknowledge that they would be more open to rural placements once they had gained more clinical experience and confidence:

‘I would like to remain with my support system for internship and train well with good supervision and resources. I would consider a rural placement for community service once I feel more confident in my abilities.’ (MBChB, female, 6th year)

Those entirely against the idea were often influenced by partner and family issues in making the decision:

‘I am married to someone with a stable job, and having to move at this point means he will likely lose his job and not be able to find work in his field of interest.’ (MBChB, female, 6th year)

### Role-players in placement decisions

Multiple role-players were identified as influencing students’ decisions around the choice of ICSP placement. The Rural Support Network (RSN), a UCT society aimed to raise awareness of rural healthcare needs among health science students, was not readily considered as a source of information by the respondents (Table 4).

**TABLE 4:** Resources used in considering internship and community service programme placement among respondents (*N* = 85).

Resources used in considering placement	*n*	%
Current or past community service workers or interns	78	91.8
Classmates	62	72.9
Clinical supervisor	41	48.2
Parents	40	47.1
Academic staff	20	23.5
Visited the proposed placement	10	11.8
Other	6	7.1
Rural support network	1	1.2

### Factors influencing decisions

Students were asked to consider multiple factors that may have influenced their decision regarding placements. These factors were grouped into two main themes: the work environment and professional support and the social and personal factors considered in decision-making (Table 5). Despite the timing of the study recruitment in 2021, no students indicated that the COVID-19 pandemic had influenced their placement decisions.

**TABLE 5:** Factors that influence the choice of internship and community service programme placement selected by students (*N* = 85).

Broad theme	Specific factors	*n*	%
Working environment professional support	The working environment within the facility	31	36.5
	Mentorship or supervision provided at the facility	27	31.8
	General safety of the facility and surrounding district	26	30.6
	Preference for field practice	13	15.3
	Access to quality of medical resources available at facility	11	12.9
	Expected workload	10	11.8
	Opportunity for personal growth	3	3.5
Social and personal factors	Access to recreational facilities nearby	30	35.3
	Proximity to home	25	29.4
	Religion and access to religious place	22	25.9
	Partner	11	12.9
	Family members Opportunity for social compact	11 9	12.9 10.6
	Travelling and transport from placement to home provinces	1	1.2
	My partner’s pregnancy	1	1.2
	My medical condition	1	1.2
	The rural allowance	7	8.2
	Children	3	3.5

When asked to elaborate on their consideration of these factors, students highlighted the following in reference to the work environment and professional support:

‘My goal for internship is to gain the skills and competencies I need for my future career, therefore I want a good working environment with supervision so that I can learn to follow the best practice but also develop my own clinical judgement.’ (MBChB, female, 6th year)

They also highlighted that certain placements were seen to be better resourced than others:

‘Would personally prefer to be in an urban setting with access to more resources, interventions, senior colleagues to get advice from.’ (MBChB, female, Male, 4th year)

When it came to issues around social and personal factors, students elaborated that:

‘Mental health is important, and I manage mine through social interaction with those around me, especially family and friends.’ (DHRS, female, 4th year)

justifying the need to be placed close to support structures. Rural placements were considered potentially unsafe and removed from close contacts: ‘Safety is very important if I’ll be staying alone as a woman’ (MBChB, female, year 6). ‘Rural placement would not allow me to be close to loved ones’ (DHRS, female, year 4), and ‘I have to apply rural but safety concerns in South Africa is making me doubt a rural placement’ (DHRS, female, 4th year).

When asked to consider the role of their UG placements on their ICSP choices, 74 (*n* = 87%) agreed that this had influenced their decision. Among MBChB students, 21 (*n* = 52%) indicated that prior exposure to the specific level of healthcare was also considered.

## Discussion

Health science students’ choices regarding ICSP placement appeared to hinge on two core areas of concern: what professional support and learning would be available and what social support they could draw on during their placements. They based their decisions largely on perceptions of diversity of clinical exposure and learning opportunities and sufficient mentorship and supervision. For MBChB students, district and tertiary care options were perceived as better sites for learning and practice. Sites perceived as underresourced and understaffed were avoided, especially for internships. A link between a supportive working environment and supervision being the preferred choice of placement emerged from students’ responses. This is supported by a review of community service for doctors that showed sufficient supervision and support to be critical factors influencing placement choice (Reid et al. 2018). Students also acknowledged the need for supervision during their ICSP to allow for further professional development. Van Stormbroek and Buchanan (2016) noted similar findings among community service occupational therapists, who reasoned that supervision contributed significantly to professional identity formation as they took up their ICSP placement.

Our findings suggest a lower uptake for primary healthcare (PHC) facilities for ICSP. This may be linked to perceptions around staffing and the range and scope of services offered at this level. However, the PHC approach forms the basis of the restructured South African health system (Van Stormbroek & Buchanan 2016). This suggests a continued need to enhance community-based clinical education in PHC facilities within undergraduate programmes. Burch and Reid (2011) argued that various challenges need to be addressed within PHC facilities in South Africa to successfully educate undergraduate FHS students and endorse clinical practice at this level of care. These include the quality of supervision, the high patient workload and the lack of space for student learning. Students may develop the necessary skills and passion for PHC practice by addressing these limitations. This may further assist in increasing students’ awareness of their professional roles in improving the health of communities at each level of care, particularly in PHC (Ramklass 2009).

Approximately half of the students were undecided or would not consider a rural placement for the ICSP. This is aligned with several studies that have demonstrated that FHS students commonly favour urban healthcare facilities (Daniels-Felix et al. 2015; McAuliffe & Barnett 2010; White & Humphreys 2014). This may threaten achieving the ICSP objectives of expanding rural healthcare service delivery. Some MBChB students indicated they would only consider moving to a rural placement after their internship once they gained clinical experience and confidence. This is perhaps appropriate as the internship programme forms the foundation for independent practice among MBChB graduates, after which they are expected to function with minimal supervision (Nkabinde et al. 2013). As the internship is 2 years and community service is only 1 year, the time needed to be spent in a rural area may be an additional consideration in the rural placement choice. Reid et al. (2018) also suggested that supervision and mentorship by experienced practitioners in rural ICSP placements would attract more professionals to these sites, strengthen the ICSP programme and promote professional development among junior colleagues.

Some evidence exists around factors influencing healthcare workers’ uptake of rural placements. Exposure to rural settings at the undergraduate level has been found to promote students’ positive perceptions of rural practice (McAuliffe & Barnett 2010; Ray et al. 2018; Van Stormbroek & Buchanan 2016) and is associated with longer-term retention of a rural workforce in both developed and developing countries (Kumar & Clancy 2020). Undergraduate faculties should consider including longitudinal rural placements as part of clinical training exposure to increase the likelihood of students considering rural practice (Burch & Reid 2011). This approach may better prepare students for prolonged rural practice and improve the uptake of rural placements for the ICSP and beyond.

Research has shown that students of rural origin commonly return to deliver service in rural areas following their studies (Dalton, Routley & Peek 2008; Kumar & Clancy 2020; McAuliffe & Barnett 2010; Reid et al. 2018), which acts as a driver for the active recruitment of students from rural backgrounds into the FHS. This may be supported by our results, where it was shown that students from rural origin showed interest in considering rural placement for the ICSP. However, it is important to note that only two respondents within our study were of rural origin. Further research should be done to confirm these findings.

Considering these aspects is important as there is less evidence on whether compulsory rural programmes improve long-term retention of staff in these posts (Kumar &Clancy 2020). While regulatory requirements like the ICSP and financial incentives can attract workers to rural settings, evidence indicates that most are likely to relocate once their agreements are completed (Esu et al. 2021).

Another potential contributor to students not considering rural placement for the ICSP is the lack of consultation with the Rural Students Network (RSN) (Table 4). The RSN is an undergraduate student society at the university that focuses on raising students’ awareness of the shortage of healthcare professionals in rural areas (Naidu & Irlam 2012). The RSN aims to enhance students’ interest in and commitment to practising in underserved rural settlements in South Africa (Naidu & Irlam 2012). However, our findings suggest insufficient utilisation of the RSN among students. Students’ knowledge of the RSN and the services provided by the society needs to be addressed.

### Social support structures

The need for social support as a factor of consideration in choosing a placement has been identified within this study. Most students identified the WC as their home province and were more likely to select the province as their first choice for ICSP placement. This is similar to the findings by Daniels-Felix et al. (2015), who showed that students commonly favour clinical learning close to their home environment where existing support structures are available. This is further supported by the 25 students reporting proximity to home as one of the most critical factors influencing ICSP placement choice (Table 3). The fact that very few participants within this sample originated from more rural provinces and areas may have contributed to the lack of uptake of rural placements. Few students seemed to express the views of Mapukata et al. (2017), who found that placements away from their home environment may significantly benefit students as they facilitate opportunities to gain confidence and independence both professionally and personally.

It is perhaps important to note that a supportive working environment within a facility could assist in creating a solid community atmosphere away from home, encouraging students to move out of their home provinces. This is supported by Rose and van Rensburg-Bonthuyzen (2015) who showed that a supportive work environment contributes to the attraction and retention of healthcare professionals in rural facilities. As rural placements comprise approximately 50% of the facilities available for the ICSP (Nkabinde et al. 2013), a supportive working environment may also influence students’ consideration of rural placements for the ICSP and improve uptake in these facilities.

Several students reported that access to recreational facilities is essential to the ICSP placement, as it promotes a good work-life balance. Rose and van Rensburg-Bonthuyzen (2015) indicated that lifestyle factors, a sense of belonging and socialisation are factors which motivated healthcare professionals to remain in rural facilities. Therefore, it is unsurprising that students considered access to nearby recreational facilities influential in their ICSP placement choice.

A common theme across the students’ responses was the need to engage outside work to socialise with other individuals, especially if relocating to a new area. In exploring physiotherapy students’ preconceptions of rural clinical placement, White and Humphreys (2014) also reported that students strongly valued access to recreational facilities for social engagement. As such, supervisors at rural ICSP placements should consider providing information on local social activities to current community service workers and interns. This may influence future ICSP workers to consider rural placements, as past community service workers and interns are commonly consulted by students when considering ICSP placement (Table 2).

### Limitations

The main limitation of the study was the small sample size, which affected the generalisation of data to the population. The study is also restricted to a single university and may not be representative of students at other universities within the province or nationally.

## Conclusion

While some differences existed between students from the MBChB and DHRS in the preferred level of healthcare, students were divided on whether they would consider a rural site for the ICSP placement. The most important factors influencing these health science students’ choice of ICSP placement were perceptions around the working environment at the facility and how this would support professional growth, as well as broader social support issues.

Undergraduate clinical exposure was investigated as a possible influencing factor for ICSP choice and seemed to have some influence on students’ choices. However, there is insufficient detail on the specific UG exposure this cohort received, so conclusions around direct links between rural or PHC exposure and ICSP choices are impossible. However, this can improve students’ readiness to work within these settings. This may lead to better uptake of these placements and assist in alleviating the service delivery challenges faced in some of these healthcare facilities. Retention of workers in rural areas in the long term remains affected by multiple issues, potentially impacting ongoing training and support of new graduates. The long-term career choices of both MBChB graduates and health and rehabilitation workers in South Africa warrant further investigation. Information surrounding the services offered by the RSN should be made more accessible to undergraduate FHS students at UCT. This may facilitate improved interest and passion for rural practice.

## Appendix 1: Questionnaires

### Section A: Demographic & Background Information

1. Are you planning to do community service/internship next year?
  □ Yes
  □ No
2. Please choose the most applicable Gender option.
  □ Male
  □ Female
  □ Other
3. Please enter your age in years
  _____________________________________
4. Please choose the most applicable marital status option.
  □ Married
  □ Single
  □ Long term relationship
  Other: _______________________________
5. Please select current degree of study at University of Cape Town:
  □ Bachelor of Medicine and Bachelor of Surgery
  □ Bachelor of Physiotherapy
  □ Bachelor of Occupational Therapy
  □ Bachelor of Speech-language Pathology
  □ Bachelor of Audiology
6. What is your home province?
  □ Western Cape
  □ Eastern Cape
  □ Northern Cape
  □ Kwa-Zulu Natal
  □ Mpumalanga
  □ Gauteng
  □ Northwest
  □ Free state
  □ Limpopo
  Other: □
7. What is your home language?
  □ English
  □ Afrikaans
  □ IsiZulu
  □ IsiXhosa Isi Ndebele
  □ siSwati
  □ Setswana
  □ Xitsonga
  □ Southern Sotho
  □ Northern Sotho
  □ Tshivenda
  Other: _______________________________
8. Which of the following languages do you speak, read or write? Please pick all
  □ English
  □ Afrikaans
  □ IsiZulu
  □ IsiXhosa
  □ IsiNdebele
  □ siSwati
  □ Setswana
  □ Xitsonga
  □ Southern Sotho Northern
  □ Sotho Tshivenda
  Other: □ _____________________________
9. Where do you currently live while you are studying?
  □ At Home
  □ University Residence
  □ Independent Residence
  □ Other
10. Based on the definitions listed below, how would you best describe the settlement area of your home environment in your home province:
  The definitions of the types of settlement areas in South Africa: -
  Rural: Living outside of urban areas, areas without access to ordinary public service such as water and sanitation –
  Peri- urban: Living in areas which surround metropolitan areas and cities. Not rural or urban in definition. –
  Urban: Areas including cities, towns and suburbs
  □ Rural
  □ Peri-urban
  □ Urban
  Other: □
11. Would you consider a rural placement for ICSP?
  □ Yes
  □ No
  □ Undecided
12. Please explain your choice
13. When considering your choice of placement, whom have you consulted or what resources have you used in deciding where to do your placement? Please tick all that apply.
  □ Classmates
  □ Current or past community service workers/interns
  □ Parents
  □ Academic staff
  □ Clinical supervisors
  □ Have visited the proposed placement.
  □ Spoken with Rural Network SA
  □ Other

### Section B: Factors influencing choice of placement for internship/community service

In the next section, use the Likert scale to rate to what extent the following factors will influence your choice of community service placement. Please link the statement below with your answer.

14) I considered this factor when making my decision for community service/internship placement:
  **Table:**
  	Strongly disagree	Disagree	Neutral	Agree	Strongly Agree
  Availability of and access to quality medical resources and staff at facility					
  Working environment at facility					
  General safety of facility and surrounding district					
  Mentorship / supervision at the facility					
  Covid 19					
  Rural allowance					
  Access to recreational facilities close to facility					
  Opportunity for social compact (i.e. being placed with another health professional)					
  Proximity To home					
  Preference for the field I want to later practice in					
  My own medical or health condition					
  My own or my partners pregnancy					
  Family contact					
  Opinion of Others					
  Religion/ access to religious places					
  Travel distance and transport to home province					
  14.1 To what extent do you agree that these individuals may have influenced your decisions around ICSP?
    **Table:**
    	Strongly disagree	Disagree	Neutral	Agree	Strongly Agree
    Partner					
    Family Members					
    Children					
    Others					
15) From the list provided in question 14, rank the three most important factors, in order of importance, that influence your choice of placement for community service/internship:
  **Table:**
  Factor	Rank 1	Rank 2	Rank 3
16) If there are any other factors which may influence your choice of placement that are not listed above, please specify below:
17) Please give reasons for your answers in questions number 15 & 16:
18) Which of the following factors from your undergraduate programme influenced your choice of placement? Please tick the most applicable:
  □ Clinical experience gained during undergraduate programme
  □ Rural exposure in undergraduate programme.
  □ Urban exposure in undergraduate programme.
  □ Exposure to the level of healthcare of my chosen placement
20) Which top 3 provinces are you planning on choosing as your choices for community service?
  **Table:**
  	First Choice	Second Choice	Third Choice
  Eastern Cape			
  Free State			
  Gauteng			
  KwaZulu- Natal			
  Limpopo			
  Mpumalanga			
  Northwest Province			
  Northern Cape			
  Western Cape			
21) At what level of healthcare would you prefer to do your ICSP placement?
  □ Primary Health Care: Community Health Centre / Clinic
  □ District Hospital
  □ Regional Hospital
  □ Tertiary Hospital
22) Please explain your preferred choice of level of healthcare:
  End of questionnaire
